# Effect of lifestyle and reproductive factors on the onset of breast cancer in female BRCA 1 and 2 mutation carriers

**DOI:** 10.1002/mgg3.191

**Published:** 2015-12-10

**Authors:** Viktoria Rieder, Mohamed Salama, Lena Glöckner, Daniela Muhr, Andreas Berger, Muy‐Kheng Tea, Georg Pfeiler, Christine Rappaport‐Fuerhauser, Daphne Gschwantler‐Kaulich, Sigrid Weingartshofer, Christian F Singer

**Affiliations:** ^1^ Department of OB/GYN and Comprehensive Cancer CenterMedical University of ViennaViennaAustria; ^2^ Department of Thoracic SurgeryOtto Wagner HospitalViennaAustria

**Keywords:** BRCA mutation, breast cancer onset, lifestyle factors, reproductive factors

## Abstract

**Background:**

The birth year‐dependent onset of breast cancer (BC) in BRCA1/2 mutation carriers suggests a risk‐modifying role for reproductive and life style factors. We therefore examined possible associations between these factors and age at diagnosis.

**Methods:**

Cox regression analysis and log‐Rank testing were used to estimate the effect of potential life style factors on the onset of BC in 197 BRCA mutation carriers.

**Results:**

Nulliparous BRCA mutation carriers developed BC earlier than those who had delivered (36.4 vs. 40.9; *P* = 0.001). Similarly, smokers and women who had used oral contraceptives experienced an earlier cancer onset (39.0 vs. 41.4; *P* = 0.05 and 39.3 vs. 44.9; *P* = 0.0001, respectively). In multivariate analysis, oral contraceptive use (HR: 1.7; *P* = 0.006) and birth cohort (< vs. ≥1965 HR: 4.5; *P* = 0.001) were associated with an earlier BC onset, while previous pregnancies led to a delay (HR: 0.2; *P* = 0.04). Mutation carriers born ≥1965 were less likely to have experienced pregnancies and more likely to have used oral contraceptives, and consequently developed BC at an earlier age (median age: 42 vs. 58; *P* < 0.0001 log‐Rank test).

**Conclusion:**

We here demonstrate that in BRCA1/2 mutation carriers the birth cohort‐associated differences in the onset of BC are profound and influenced by reproductive factors.

## Introduction

Breast cancer (BC) is the most common malignancy in women with a worldwide incidence rate of 11.9% (Ferlay et al. 2014). While the majority of BC cases are sporadic, approximately about 5–10% are estimated to be of genetic predisposition. Of these, 40% are due to mutations in either of the two tumor suppressor genes BRCA (BReast CAncer gene) 1 and 2 (Miki et al. 1994; Narod 2002; Palma et al. 2006). Female BRCA1 and BRCA2 mutation carriers face a high lifetime risk of developing BC and ovarian cancer (OC). The cumulative risk for developing BC until the age of 70 has been estimated to be up to 87% for BRCA1 mutation carriers (Ford et al. 1994; Antoniou et al. 2000, 2003; Risch et al. 2001; Mavaddat et al. 2013), and up to 84% for BRCA2 mutation carriers (Ford et al. 1998; Thorlacius et al. 1998; Warner et al. 1999; Antoniou et al. 2000; Risch et al. 2001). However, risk estimates for developing BC show considerable divergence. They vary in respect of the populations studied as well as in consequence to different study designs used. Furthermore, variations in the risk of developing BC have been reported in relation to the position of the mutation within the genes (Thompson and Easton 2001, 2002). In addition, studies have recently demonstrated that BRCA germline mutation carriers who belong to a younger birth cohort face a higher risk to develop BC, and that the onset of BC is significantly influenced by the year of birth (King et al. 2003; Kroiss et al. 2005; Evans et al. 2008; Tea et al. 2014), thereby indicating a risk‐modifying role for reproductive and life style factors.

Genetic counseling for BRCA1 and BRCA2 mutation carriers is offered worldwide, and risk‐reducing strategies such as prophylactic surgery are commonly considered by the majority of affected women. Since female BRCA1 or 2 mutation carriers have a particularly high risk to develop BC at a young age, it is highly important to predict the individual onset of BC more precisely. An information on potential risk modifiers and their contribution to BC onset will consequently allow for a personalized strategy for BC prevention (Narod 2006).

We have therefore examined possible associations between potential birth year, reproductive and lifestyle factors and biological risk factors, birth cohorts, and the onset of BC in BRCA1 and BRCA2 mutation carriers.

## Materials and Methods

### Ethical compliance

The study was approved by the local IRB board.

### Study population

Genetic testing for BRCA1 and BRCA2 has been offered to women with a familial BC and/or OC background at the Vienna General Hospital since 1995. Since then, 701 individuals with a mutation in BRCA1, 325 individuals with a mutation in BRCA2, and 9 individuals with a mutation in both genes, BRCA1 and BRCA2, have been identified. Eligible study subjects were women with a confirmed mutation in either BRCA1 or BRCA2, who had developed BC and who had completed a questionnaire on potential lifestyle and reproductive risk factors either at the time of BC diagnosis or after BC diagnosis. Questionnaires were usually sent out and completed by the patient. In some instances, the questionnaire was completed by the patient with assistance by a certified study nurse, if required. Date of birth, mutation status, parity, smoking and alcohol intake history, height, and weight were obtained by reviewing the patients′ medical records. Weight and height were obtained at BC diagnosis as part of the hospital routine procedure. Subjects were at least 18 years old, and had either been seen at the Department of OB/GYN at the Vienna General Hospital, or in one of the 70 affiliated outpatient clinics throughout Austria. In order to be eligible for genetic counseling, they had to meet local selection criteria which have previously been described and had to sign an informed consent before blood sampling for molecular analysis was taken (Tea et al. 2014).

### Statistical analysis

Data was analyzed retrospectively, and for statistical analyses, the SPSS software package (SPSS, Ehningen, Germany) was used. Women who had undergone prophylactic surgeries, such as prophylactic bilateral salpingo oophorectomy (PBSO) or bilateral prophylactic mastectomy (BPM), as well as women who had not developed BC at time of analysis (November 2013) were censored. Univariate and multivariate Cox regression analyses were used to estimate the effect of potential risk factors on the onset of BC. BC risks depending on birth cohorts (before/after 1965) were estimated using Kaplan–Meier analysis, log‐Rank test, and Student′s *t*‐test. Two‐sided *P*‐values ≤ 0.05 were considered to be statistically significant.

## Results

### Reproductive and life style factors and BC onset

BRCA status, reproductive and lifestyle factors were available in 366 patients. The median birth year in the overall study population was 1965. BRCA1/2 mutation carriers, who had never been pregnant, developed BC at a younger age than those who had delivered at least once (36.4 vs. 40.9; *P* = 0.001 Student′s *t*‐test). Similarly, women who had used oral contraceptives, or had smoked, developed BC earlier than never users (39.3 vs. 44.9 years; *P* < 0.0001; Student′s *t*‐test) or women who had never smoked (39.0 vs. 41.4 years; *P* = 0.05; Student′s *t*‐test). No association with an earlier BC onset was observed for breastfeeding and alcohol consumption (data not shown).

Univariate analysis revealed that women who had been pregnant at least once developed BC at a later age than those who had never been pregnant (HR: 0.51; *P* = 0.001). Similarly, the more full‐term pregnancies they had, the longer their BC onset was delayed (HR: 0.81; *P* = 0.005) (Table 1). By contrast, women who reported prior or concurrent use of oral contraceptives developed BC at a younger age (HR: 1.93; *P* = 0.002), but the later they started the use of oral contraceptives, the later they developed BC (HR: 0.87; *P* < 0.001). Ever having smoked was also associated with an earlier BC onset (HR: 1.5; *P* = 0.009) and so was birth cohort after 1965 (HR: 5.08; *P* < 0.0001). No association between BC onset and age at birth of first child, breast‐feeding, duration of breast‐feeding, duration of oral contraceptive use, age at last use of oral contraceptives, alcohol intake, and duration of smoking was observed in univariate analysis.

**Table 1:** Univariate and multivariate analysis: influence of potential risk factors on breast cancer onset (significant HR in bold)

Parameter	HR (CI) ‐ univariate	*P*‐value	Adjusted HR (CI) ‐ multivariate	*P*‐value
BRCA mutation	0.75 (0.6–1.01)	0.06		
Parity (never/ever pregnant)	**0.51** (0.3–0.75)	**0.001**	2.3 (0.09–60.8)	0.6
Number of full‐term pregnancies	**0.81** (0.7–0.94)	**0.005**	**0.15** (0.02–0.9)	**0.04**
Age at birth of first child	1.01 (0.97–1.04)	0.7		
Breast‐feeding	0.7 (0.45–1.08)	0.11		
Duration of breast‐feeding	1.01 (0.99–1.03)	0.2		
Oral contraceptive use	**1.93 (**1.29–2.88)	**0.002**	**1.7** (1.1–2.05)	**0.006**
Duration of oral contraceptive use	1.00 (0.99–1.00)	0.18		
Age at first use of oral contraceptives	**0.87** (0.83–0.92)	**0.0001**	1.03 (0.8–1.3)	0.8
Age at last use of oral contraceptives	0.96 (0.96–1.01)	0.12		
Alcohol (never/ever)	1.16 (0.86–1.57)	0.32		
Smoking (never/ever)	**1.50** (1.10–1.90)	**0.009**	1.1 (0.23–4.6)	0.97
Duration of smoking	1.01 (0.99–1.02)	0.29		
Birth <1965	**5.08** (3.6–7.18)	**0.0001**	**3.7** (2.4–5.7)	**0.001**

In multivariate analysis (see Table 1), oral contraceptive use (HR: 1.7; *P* = 0.006), and date of birth ≥1965 (HR: 4.5; *P* = 0.001) were associated with an earlier BC onset. Conversely, the more full‐term pregnancies a woman had experienced, the longer her BC onset was delayed (HR: 0.15; *P* = 0.04).

### Birth cohort and BC onset

**Figure 1:**
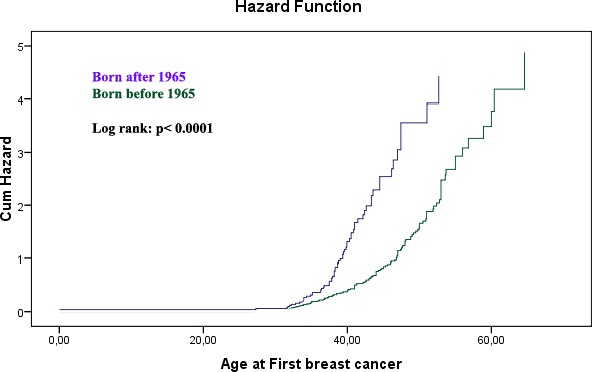
Birth cohort and age at onset of breast cancer in *BRCA1* and *2* germline mutation carriers.

Mutation carriers born in or after 1965 developed BC at an earlier age when compared to those who were born before 1965 (Fig. 1; *P* < 0.0001, log‐Rank test). The median age of BC diagnosis for women who were born ≥1965 was 42 as compared to 58 years for women born <1965 was 58 years.

When we looked at differences in lifestyle and reproductive factors in women born before versus after 1965, we found that women in the earlier birth cohort were significantly more likely to have experienced one or more pregnancies (*P* < 0.0001, Fisher′s exact test), and to have undergone at least one delivery (*P* < 0.0001). As expected, women born before 1965 were significantly younger at the time of their first delivery (*P* < 0.0001). Women born before 1965 were less likely to have used oral contraceptives (*P* = 0.004), and to have used HRT (*P* ≤ 0.0001). They were also significantly less likely to be obese (*P* = 0.0410, chi‐square test). The two birth cohorts did not differ significantly when smoking history, alcohol consumption, history of breast‐feeding, and age at menarche were compared. (Table 2).

**Table 2:** Patient characteristics in women born <1965 ≥1965 (significant differences in bold)

Birth cohort	<1965	≥1965	*P*‐value
BRCA‐mutation			
BRCA1	135 (67.2%)	123 (74.5%)	
BRCA2	66 (32.8%)	42 (25.5%)	*P* = 0.1352
History of pregnancies			
No	20 (10.0%)	59 (36.0%)	
Yes	181 (90.0%)	105 (64.0%)	***P*** ** < 0.0001**
Number of deliveries	1.9 ± 1	1.08 ± 0.9	***P*** ** < 0.0001**
History of breastfeeding			
No	21 (12.0%)	17 (17.2%)	
Yes	153 (88.0%)	82 (82.8%)	*P* = 0.2764
Max BMI weight cohort			
BMI <18	0 (0.0%)	1 (0.9%)	
BMI 18–25	48 (39.0%)	64 (54.2%)	
BMI 25–30	46 (37.4%)	39 (33.1%)	
BMI >30	29 (23.6%)	15 (12.7%)	***P*** ** = 0.0410**
Oral contraceptive use			
Never	91 (37.4%)	42 (21.4%)	
Ever	152 (62.6%)	154 (78.6%)	***P*** ** = 0.0004**
HRT use			
Never	140 (73.7%)	141 (92.8%)	
Ever	50 (26.3%)	11 (7.2%)	***P*** ** < 0.0001**
Alcohol (never/ever)			
Never	72 (36.2%)	48 (29.1%)	
Ever	127 (63.8%)	117 (71.9%)	*P* = 0.1790
Smoking (never/ever)			
Never	99 (49.3%)	84 (50.1%)	
Ever	102 (50.7%)	81 (49.9%)	*P* = 0.8336
Age at menarche	12.9 ± 1.6	12.9 ± 1.4	*P* = 1.0000
Age at birth of first child	23.9 ± 4.3	26.4 ± 4.4	***P*** ** < 0.0001**

## Discussion

This study investigated the influence of potential risk factors on the disease onset in a cohort of Austrian BRCA1/2 mutation carriers who had developed BC. In addition, we analyzed whether longitudinal changes in reproductive and life style factors could explain the earlier onset in BRCA mutation carriers who belonged to a younger birth cohort.

We identified reproduction‐related parameters to be the most important risk factors related to earlier BC onset. Our findings are in line with several observations in non‐high‐risk populations, where a history of pregnancies has consistently shown to be related to a significantly lower BC risk (Kelsey et al. 1993; Russo et al. 2005). Previous studies investigating the influence of pregnancies on the BC risk in BRCA1/2 mutation carriers have, however, yielded inconsistent results: while some authors reported no association with all or even an increased BC risk, with an elevated risk for each additional pregnancy (Jernstrom et al. 1999; Gronwald et al. 2006), others have described a protective effect and a risk reduction for each additional birth (Andrieu et al. 2006; Antoniou et al. 2006; Milne et al. 2010). In our study, having undergone at least one full‐term pregnancy increased the age at disease onset in a mutation carrier by 4.5 years when compared to a nullipara. Consistent with these results, we also found that the more full‐term pregnancies a women had experienced, the longer her BC onset was delayed.

Furthermore, our findings suggest that BRCA1/2 mutation carriers who use oral contraceptives develop BC at a younger age. Whether exogenous estrogens, such as oral contraceptives, modify the BC risk in BRCA1/2 mutation carriers is a controversial topic: while some studies suggest that oral contraceptives may increase the BC risk among BRCA1/2 mutation carriers, others reported only little or no influence of oral contraceptives on the BC risk (Narod et al. 2002; Gronwald et al. 2006; Brohet et al. 2007; Lee et al. 2008). A large study conducted by Brohet et al. (2007) found that ever use of oral contraceptives as well as longer duration of oral contraceptive use were not only associated with an increasing BC risk, but also with an earlier onset.

Studies investigating the effect of smoking on BC risk in the general population have yielded divergent results (Hamajima et al. 2002; Terry and Rohan 2002; Al‐Delaimy et al. 2004; Reynolds et al. 2004, 2006), and several pathophysiological mechanisms have been discussed in order to explain either an increased or decreased BC risk in smokers in the respective studies: the carcinogenic effects of metabolites in cigarette smoke were thought to explain an increase (IARC, 1986; el‐Bayoumy 1992; Morris and Seifter 1992; Hoffmann et al. 2001), while a reduction in estrogen exposure due to a lower body fat and an earlier menopause were hypothesized to be responsible for a decrease in BC risk (MacMahon et al. 1982; Lesko et al. 1985; Baron et al. 1990; Reynolds et al. 2004). Studies investigating the effect of smoking and BC risk in the considerably smaller group of BRCA1/2 mutation carriers have again lead to different outcomes: some reported no association at all (Gronwald et al. 2006; Nkondjock et al. 2006), others found elevated risk (Lecarpentier et al. 2011), while again others even described a protective association between smoking and BC (Colilla et al. 2006). We were unable to detect an overall association between smoking and BC onset in multivariate analysis, but nevertheless observed that in BRCA mutation carriers, smokers developed BC significantly earlier.

Previous studies investigated the influence of birth cohorts on the BC risk and BC onset in BRCA1/2 mutation carriers (King et al. 2003; Kroiss et al. 2005; Evans et al. 2008; Tea et al. 2014). Two of these trials were conducted at our institution and revealed that a younger birth cohort correlates with a higher BC risk and earlier BC onset in BRCA1 and 2 mutation carriers (Kroiss et al. 2005; Tea et al. 2014). This strongly suggests a role for modifying risk factors which may have changed over time. In our overall study population, the median birth year was 1965, and women born before that year developed BC on average 16 years later than those born in 1965 or later. This difference was paralleled by differences in reproductive factors such as number of pregnancies, parity, age at first delivery, which have all been subject to considerable changes in the overall population over the last decades.

While women born before 1965 were significantly younger at the time of their first delivery, this did not have an effect on the age at BC onset. Our data are therefore somewhat contradictory to findings from the general population which consistently describe that delivery at a younger age provides a profound and sustained protection from BC (Kobayashi et al. 2012).

The increase in oral contraceptive use over the last decades is closely linked to the decline in pregnancies during this time period and is reflected in our study population. While oral contraceptives have been shown to protect from OC, they are also linked to a small and transient, but nevertheless significant increase in BC (Moorman et al. 2013). It is thus possible that the unfavorable effect of oral contraceptives in the early development of BC in mutation carriers is mainly indirect, through the prevention and delay of pregnancies, rather than through a direct interaction.

We did not observe differences in the behavioral pattern regarding smoking and alcohol consumption in the two birth cohorts. When we, however, looked at HRT intake in women born before and after 1965, we did find significant differences. This observation is not surprising since mutation carriers in the younger birth cohort group were predominantly premenopausal at the time of analysis. HRT intake was, however, not associated with BC onset, possibly due to the relatively small number of individuals (data not shown).

Taken together, we here demonstrate that in BRCA1 and 2 mutation carriers the birth cohort‐associated differences in the onset of BC are profound and should therefore be accounted for in genetic counseling. We further hypothesize that the onset of BC in this population can be influenced by reproductive factors such as the number of pregnancies and the use of oral contraceptives.

## Conflict of Interest

The authors declare no conflict of interest. The study has been approved by the local ethics committee.
